# Dynamic stability during level walking and obstacle crossing in children aged 2–5 years estimated by marker-less motion capture

**DOI:** 10.3389/fspor.2023.1109581

**Published:** 2023-04-06

**Authors:** Kohei Yoshimoto, Hiroki Mani, Natsuki Hirose, Takaki Kurogi, Takumi Aiko, Masahiro Shinya

**Affiliations:** ^1^ Graduate School of Humanities and Social Sciences, Hiroshima University, Higashi-Hiroshima, Japan; ^2^ Faculty of Welfare and Health Science, Oita University, Oita, Japan; ^3^ Graduate School of Welfare and Health Science, Oita University, Oita, Japan

**Keywords:** margin of stability (MOS), preschoolers, obstacle advoidance, balance, adaptive locomotion, development

## Abstract

In the present study, dynamic stability during level walking and obstacle crossing in typically developing children aged 2–5 years (*n* = 13) and healthy young adults (*n* = 19) was investigated. The participants were asked to walk along unobstructed and obstructed walkways. The height of the obstacle was set at 10% of the leg length. Gait motion was captured by three RGB cameras. 2D body landmarks were estimated using OpenPose, a marker-less motion capture algorithm, and converted to 3D using direct linear transformation (DLT). Dynamic stability was evaluated using the margin of stability (MoS) in the forward and lateral directions. All the participants successfully crossed the obstacles. Younger children crossed the obstacle more carefully to avoid falls, as evidenced by obviously decreased gait speed just before the obstacle in 2-year-olds and the increased in maximum toe height with younger age. There was no significant difference in the MoS at the instant of heel contact between children and adults during level walking and obstacle crossing in the forward direction, although children increased the step length of the lead leg to a greater extent than the adults to ensure base of support (BoS)-center of mass (CoM) distance. In the lateral direction, children exhibited a greater MoS than adults during level walking [children: 9.5%, adults: 6.5%, median, *W* = 39.000, *p* < .001, rank-biserial correlation = −0.684]; however, some children exhibited a smaller MoS during obstacle crossing [lead leg: −5.9% to 3.6% (min–max) for 4 children, 4.7%–6.4% [95% confidence interval (CI)] for adults, *p* < 0.05; trail leg: 0.1%–4.4% (min–max) for 4 children, 4.7%–6.4% (95% CI) for adults, *p* < 0.05]]. These results indicate that in early childhood, locomotor adjustment needed to avoid contact with obstacles can be observed, whereas lateral dynamic stability is frangible.

## 1. Introduction

The period between the onset of independent walking and the age of 4–5 years is crucial for the reorganization of gait patterns in children following the rapid maturation of body size and the sensorimotor system. Previous studies have reported that the gait patterns of steady walking rapidly develop 3–6 months after the onset of independent walking (1–3). Subsequently, step length and step speed (normalized to body size) become adult-like at approximately 4–5 years of age (4–8). However, in addition to level walking, children need to locomote in a complex environment, despite their immature gait function. The difficulty that preschool children face in adapting to external objects during locomotion is accentuated by the fact that among 750 children under 12 years of age with craniofacial injuries from falls, preschool children accounted for 72% of trips; colliding with an obstacle is a cause of tripping (9).

Obstacle crossing is an important adaptive locomotion skill for children, as they are often required to cross obstacles, such as toys and furniture, that expose them to a higher risk of falling than adults. To safely cross obstacles, individuals must exhibit proper foot clearance and high stability of the center of mass (CoM). Using a large foot clearance and slower walking speed to cross obstacles is a well-known compensatory strategy (10–12); elderly people who are at high risk of falling try to minimize the risk of obstacle contact and control forward momentum. In contrast, Yamagata et al. (13) reported that maximum foot elevation was correlated with low control of the CoM position in the frontal plane. Several previous studies have reported that foot clearance is larger in younger children (14, 15), which may induce lateral instability of the CoM.

The margin of stability (MoS) is an index used to evaluate dynamic stability during walking (16). A comprehensive literature search revealed that the stability of the CoM during obstacle crossing in children has been assessed in healthy children aged 5–16 years (17), children with cerebral palsy (18), and children with a developmental coordination disorder (19); in these studies, the CoM position and CoM velocity were evaluated separately. In contrast, the MoS incorporates not only the CoM position but also the CoM velocity with respect to the base of support (BoS). During level walking, preschool children exhibit a large CoM displacement; specifically, children aged 1–3 years show greater lateral CoM displacement during the one-leg stride than adults (20, 21), and even when walking speed was controlled, the CoM exhibited more lateral sway in younger children (20). Paradoxically, younger children exhibited a large lateral MoS during level walking, which indicates that the CoM is unlikely to pass beyond the lateral edge of the BoS of the single support foot in the swing phase by compensating for the large CoM sway with wide step widths (6). However, it is unclear whether preschool children exhibit a large MoS during obstacle crossing as well as level walking.

Previous studies on obstacle crossing have rarely focused on typically developing children aged 2–5 years. Children are able to step over obstacles independently at approximately 2 years of age. Dominici et al. (22) reported that toddlers under 2 years of age with less than 2 weeks of walking experience struggle to cross an obstacle without support. Although children over 2 years of age could cross obstacles independently, the obstacle-crossing tasks were challenging, as evidenced by the fact that these children stopped for a few seconds before crossing obstacles (23). Cappellini et al. (23) conducted the only study that measured a wide range of obstacle-crossing behaviors in young children (between the ages of 2 and 12); however, developmental trends in the characteristics of obstacle-crossing behavior in preschool children have yet to be elucidated.

The purpose of this study was to investigate dynamic stability during level walking and obstacle crossing in preschool children (aged 2–5 years) using marker-less motion capture. We compared the MoS and typical gait parameters during obstacle crossing between children and young adults. Moreover, we assessed developmental changes in obstacle-crossing behavior in preschool children. We assumed that children would show more dynamic stability during level walking and less dynamic stability during obstacle crossing than adults.

## 2. Methods

### 2.1. Participants

To be included, children had to be 2–5 years old, and adults had to be over 18 years old. The exclusion criteria were as follows: free from musculoskeletal or neurological disorders that might affect walking (all participants) and free from neuromuscular diseases or attentional deficits (children; according to parent and teacher reports). Electronic advertisements and paper flyers were used to recruit participants. A total of 13 healthy children aged 2–5 years (5 females/8 males, height = 100.6 ± 1.03 cm, weight = 16.8 ± 3.2 kg; including three 2-year-olds, two 3-year-olds, three 4-year-olds, and five 5-year-olds) and 19 healthy young adults (11 females/8 males, age = 21.9 ± 3.2 years, height = 164.9 ± 8.9 cm, weight = 56.9 ± 9.3 kg) were included in this study. Informed consent was obtained from the participants and the parents of each child before the start of the experiment, and informed assent was obtained from all children. The study was conducted in accordance with the Declaration of Helsinki and approved by the local ethics committee of Oita University Faculty of Welfare and Health Science (approval number: F200016).

### 2.2. Experimental protocols

**Figure 1:**
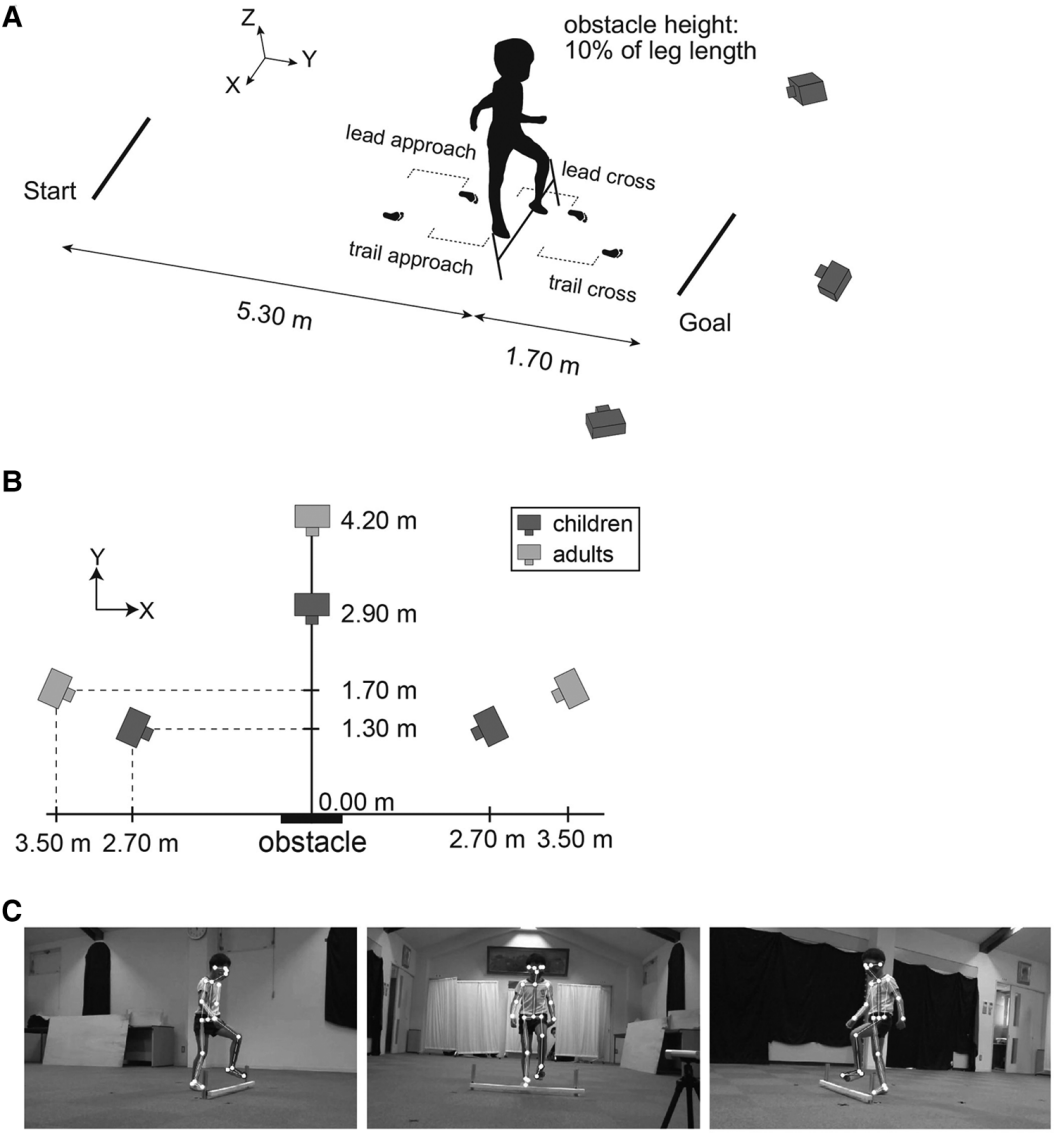
Experimental setup and overview of estimating body feature points by OpenPose. (**A**) Obstacle-crossing tasks were recorded by three RGB video cameras placed in front [perspective in (**C**), middle image] and diagonally in front [perspective in (**C**), left and right images] of an obstacle. The data from a total of 4 steps, including two steps before crossing the obstacle (called the lead approach step and trail approach step) and two steps during crossing the obstacle (called the lead cross step and trail cross step), were analyzed. (**B**) Camera positions for the children were closer to the obstacle than those for the adults. (**C**) OpenPose was applied to the three recording videos to estimate the 2D coordinates of the 25 body landmarks; these coordinates were transformed into 3D coordinates using a direct linear transformation (DLT).

The participants walked at a self-selected pace along a 7.00-m unobstructed and obstructed walkway. The height of the obstacle was normalized to 10% of leg length, which was determined based on previous studies that used 16% and 23% leg length for 0.8–1.3-year-olds (22), 4%–8% leg length for 1.8–12.0-year-olds (23), and 20%–25% leg length for 4- and 6-year-olds (15). The obstacle was placed 5.30 m from the starting position; thus, the children and the adults took at least 9 and 6 steps before they encountered the obstacle, respectively (Figure 1A). We used a hurdle-like obstacle composed of a Styrofoam bar (800 mm wide × 15 mm square) placed across an aluminum frame. The participants completed at least 5 trials. However, children occasionally deviated from the specified instructions by engaging in movements such as jumping or running. In cases where fewer than 5 trials were deemed available for analysis at the 5th trial, up to 2 additional trials were conducted at the discretion of experimenters. As a result, the numbers of trials available for analysis in the children were 2–5 trials. In the obstacle-crossing task, no instructions were given as to which leg should be used first when crossing the obstacle. Before the experimental trials, participants were allowed up to 3 practice trials for each walking task to familiarize themselves with the experimental environment. We measured participant height, weight, leg length, pelvic width, and foot length.

### 2.3. Measurements

Gait motion was captured by three RGB cameras (SONY, FDR-AX45BC) (resolution: 1,980 × 1,080 dpi, frame rate: 120 fps). One camera was placed in front of the obstacle, and the other cameras were placed diagonally in front of the obstacle on the left and right sides. The accuracy of marker-less motion capture depends on the size of the whole body reflected in the view. Because children have smaller body sizes than adults, the camera positions for the children were closer to the participants than those for the adults (Figure 1B). The three recording videos were time-synchronized to the illumination of an LED emitted each trial. The 2D coordinates of the body landmarks were estimated by using OpenPose (24) version 1.7.0, a marker-less motion-capture algorithm (Figure 1C). A total of 25 body landmarks were obtained: nose, neck, middle hip, left and right eyes, ears, shoulders, elbows, wrists, hips, knees, ankles, big toes, small toes, and heels. The 2D coordinates of these body landmarks were converted to 3D coordinates using direct linear transformation (DLT). Although OpenPose had better results in slower movements such as walking compared to jumping and throwing movements, it is also known that incorrectly detected joints produced large measurement errors (e.g., detecting the left knee as the right knee) (25). In the present study, the possibility of such a large measurement error was minimized by excluding outliers by visual inspection. The global coordinate system was configured with a positive *x*-axis in the right direction, a positive *y*-axis in the forward direction, a positive *z*-axis in the upward direction, and an origin based on the location of the obstacle.

### 2.4. Data analysis

The 3D raw kinematic data were smoothed using a zero-lag second-order low-pass digital Butterworth filter. The cutoff frequency was determined for each time series by residual analysis (26). The range of the selected cutoff frequency was 1.1–7.8 Hz. The timing of the heel contact was defined as when the velocity of the heel position dropped below 10% of the peak velocity of the heel position. The timing of toe-off was defined as when the velocity of the big toe position exceeded 10% of the peak velocity of the big toe position.

**Figure 2:**
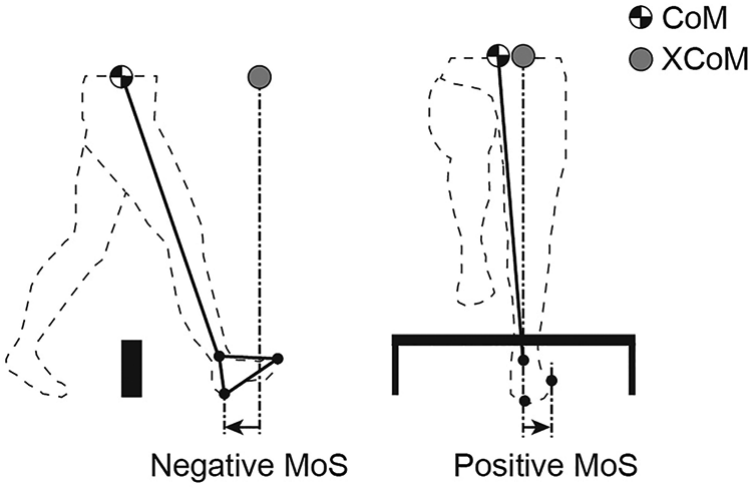
Definition of the margin of stability (MoS). The extrapolated center of mass (XCoM) was derived from the position and velocity of the center of mass (CoM). MoS in the anteroposterior (AP) direction was defined as the heel position minus the XCoM at the time of heel contact. The MoS in the mediolateral (ML) direction was defined as the minimum distance from the small toe or heel position minus the XCoM. A positive MoS indicates dynamic stability. A negative MoS indicates that the walker would theoretically need an extra step to ensure that the CoM remained within the base of support (BoS).

The position of the CoM was estimated by a 14-body-segment model according to anthropometric data from children (27) and adults (26). The CoM velocity was calculated as the first derivative of the CoM position using a 3-point differentiation. The extrapolated center of mass (XCoM) was calculated following the equation introduced by Hof et al. (16):

$$XCoM=x+\frac{\dot{x}}{\omega_{0}}$$

where *x* is the position of the CoM, *ẋ* is the velocity of the CoM, and *ω*_0_ is the eigenfrequency of the pendulum in the model, calculated as

$$\omega_{0}=\sqrt{\frac{g}{l}}$$

where *g* = 9.81 m/s^2^ is the gravitational acceleration and l is the pendulum length, calculated as 1.24 × leg length (16). The MoS was calculated in the anteroposterior (AP) and mediolateral (ML) directions (Figure 2). The MoS in the AP axis was defined as the difference from the anterior edge of the base of support (BoS) to the XCoM in the AP axis at the timing of heel contact of the lead and trail legs, and the timing was determined following a previous study (28) (The results of AP MoS at the timing of toe-off are shown in a Supplementary Material). The anterior edge of the BoS was defined by the heel position. The MoS in the ML axis was defined as the minimum distance from the lateral edge of the BoS to the XCoM in the ML axis during the swing phase of the lead and trail legs, measured from toe-off to heel contact. The lateral edge of the BoS was defined as the most lateral position between the small toe and heel marker; if the stance feet turned in (pigeon toe), the lateral edge of the BoS was defined by the heel position. A positive MoS indicates a stable state (i.e., the CoM returns to its current position when the XCoM is positioned within the BoS); a positive AP MoS means that the CoM will not fall forward, and a positive ML MoS means that the CoM will not fall laterally. The instantaneous BoS-CoM distance and CoM velocity given the MoS were also calculated. In the level-walking task, the MoS, BoS-CoM distance and CoM velocity were calculated as the average of 2 strides.

We calculated the vertical foot clearance, maximum toe height, and swing time of the lead and trail leg during obstacle crossing. The vertical foot clearance was defined as the vertical distance between the big toe and the obstacle when the big toe was directly above the obstacle. Additionally, the step length, step width and step speed were assessed during the obstacle-crossing and level-walking tasks. During the obstacle-crossing task, a total of 4 steps [two steps before crossing the obstacle (the lead approach step and the trail approach step) and two steps during obstacle crossing (the lead cross step and the trail cross step)] were analyzed (see Figure 1A). During the level-walking task, the average of these gait parameters over 4 steps was calculated. The step length and step width were defined as the AP and ML distance, respectively, between two heel positions at the timing of the heel contact. The step speed was defined as the step length divided by the step duration. To assess the changes in gait pattern from level walking to obstacle crossing, we calculated the change ratio of obstacle crossing to level walking for the step length, step width, and step speed, similar to the analysis by Corporaal et al. (29). These spatial, temporal and velocity parameters were normalized by leg length, the square root of leg length divided by gravity and the square root of gravity times leg length, respectively.

### 2.5. Statistical analysis

Because of the large variability among children, the Mann‒Whitney *U*-test was used to determine whether there was a significant difference between the children and adult groups. Additionally, a one-sample Student's t test was performed to clarify whether each child significantly differed from the adult group. The significance level was set at *α* < 0.05.

## 3. Results

### 3.1. Gait parameters during level walking

**Figure 3:**
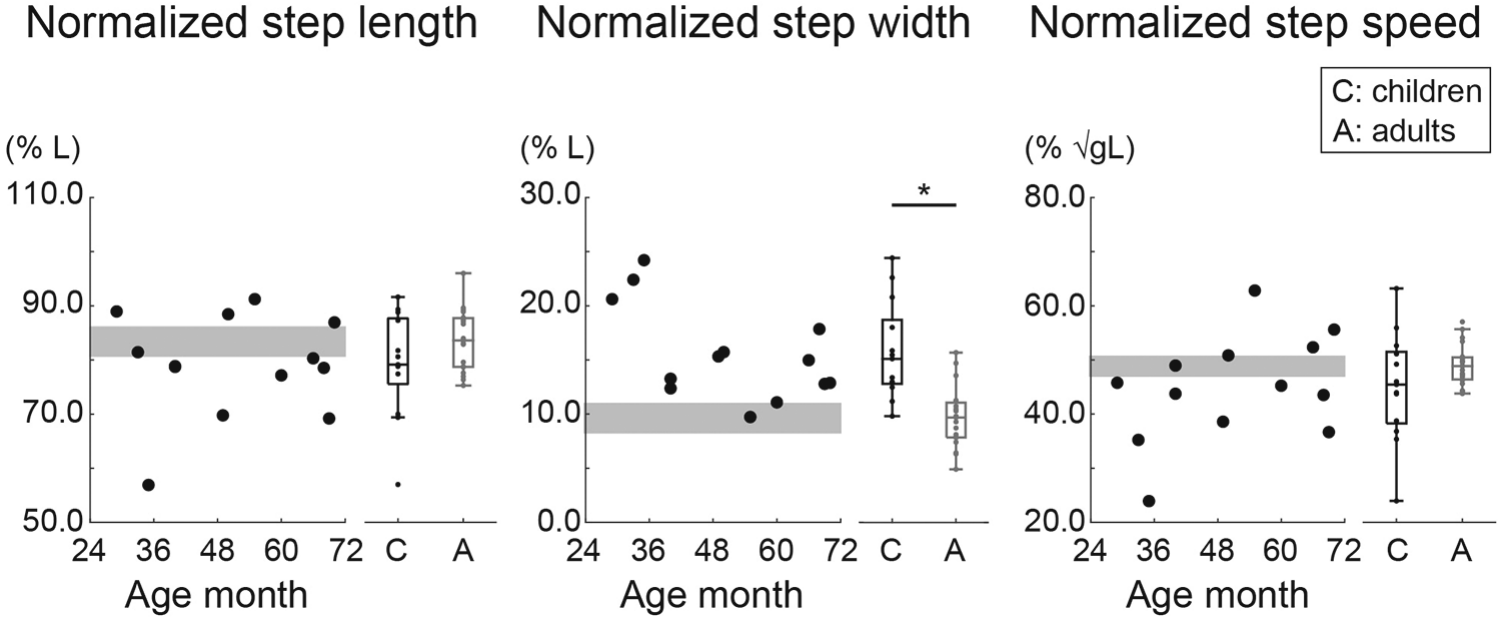
Normalized step length, step width, and step speed during level walking. The step length and step width were normalized to leg length (*L*), and the step speed was normalized to the root square of gravity (*g*) × *L*. Circles indicate the mean within-participant value. The gray area shows the 95% confidence interval (CI) of the mean of adults. * Indicates *p* < 0.05.

Figure 3 shows the normalized step length, step width, and step speed during level walking. The mean value of the normalized step length was 75.8% for the 2-year-olds, 78.8% for the 3-year-olds, 83.2% for the 4-year-olds, 78.4% for the 5-year-olds, and 83.3% for the adults. The mean value of the normalized step width was 22.4% for the 2-year-olds, 12.8% for the 3-year-olds, 13.6% for the 4-year-olds, 13.9% for the 5-year-olds, and 9.6% for the adults. The mean value of the normalized step speed was 35.0% for the 2-year-olds, 46.4% for the 3-year-olds, 50.8% for the 4-year-olds, 46.7% for the 5-year-olds, and 48.8% for the adults. Although there were no significant differences in the normalized step length and step speed between children and adults, most of the children (8/13) took significantly shorter and slower steps than the adults (*p* < 0.05). Specifically, a 2-year-old showed an extremely small step length and step speed. In contrast, children took significantly wider steps than adults (*W* = 26.000, *p* < .001, rank-biserial correlation = −0.789). In particular, children aged 2 years had a greater step width than children aged 3–5 years.

### 3.2. Gait styles during obstacle crossing in preschool children and adults

**Figure 4:**
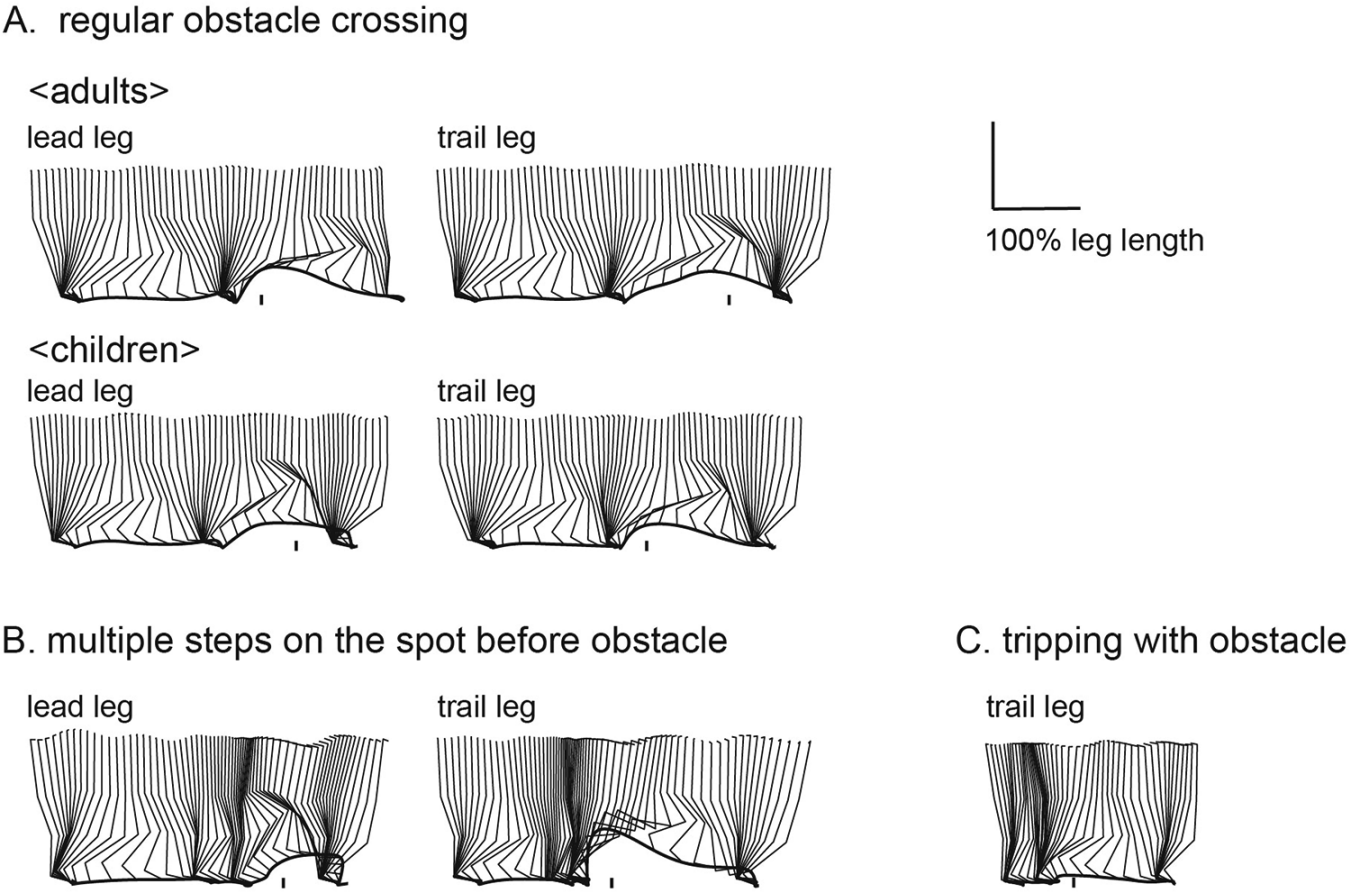
Examples of sagittal stick pictures normalized to leg length during obstacle crossing. Most children aged 2–5 years were able to clear an obstacle set to a height of 10% of leg length as well as adults [(**A**), top: adult, 20 years old; bottom: child, 5.7 years old]. Children varied in obstacle-crossing behavior, with one taking several steps on the spot before crossing an obstacle [(**B**): child, 2.4 years old] and one bringing the trail leg into contact with the obstacle [(**C**): child, 2.9 years old].

Figure 4 shows representative trials of adults and children. In the adult group, all participants successfully crossed the obstacle without hitting it in any trial (Figure 4A). In the child group, different obstacle-crossing performances were observed. Children aged 2–5 years usually successfully crossed the obstacle (Figure 4A). However, a girl aged 2.4 years took multiple steps on the spot before the obstacle for 1 s and then crossed the obstacle in 1 trial (Figure 4B). Another girl (aged 2.9 years) had one failed trial in which her trail leg contacted the obstacle (Figure 4C): except for a single girl (aged 2.9 years), twelve out of thirteen children successfully crossed the obstacle without collision with the obstacle. These 2 trials were excluded from the analysis.

### 3.3. Margin of stability

**Figure 5:**
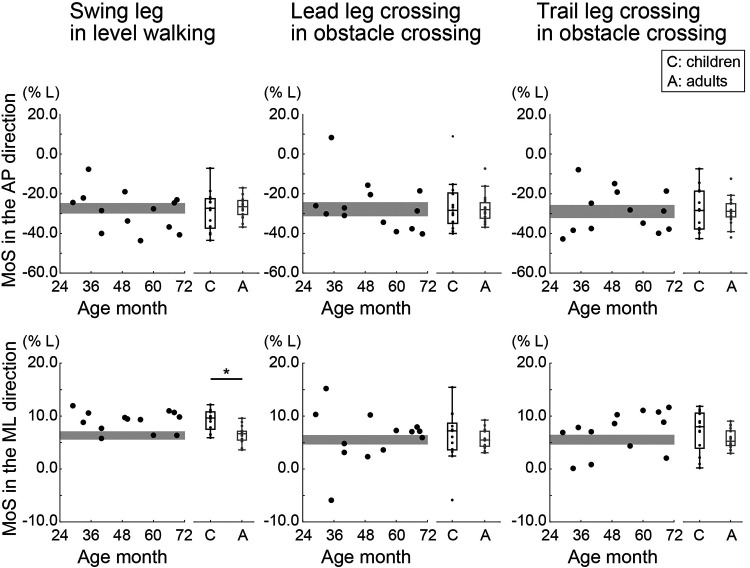
Normalized margin of stability (MoS) in the anteroposterior (AP) and mediolateral (ML) directions during level walking and crossing an obstacle (with the lead and trail leg, separately). The MoS was normalized to leg length (*L*). Circles indicate the mean value of within participants. The gray area shows the 95% confidence interval (CI) of the mean of adults. * Indicates *p* < 0.05.

Figure 5 shows the normalized MoS in the AP and ML directions for both children and adults during level walking and obstacle crossing. Along the AP axis, there were no significant differences in the normalized MoS at the instant of heel contact between the children and adults during level walking and lead and trail leg crossing the obstacle (*p* > 0.05). Children exhibited large individual differences, showing normalized MoS values equal to, larger than, or smaller than those of adults. Specifically, a positive normalized MoS value was observed after the lead leg crossed the obstacle in a child aged 2.9 years.

Along the ML axis, the normalized MoS during level walking was significantly greater in children than in adults (*W* = 39.000, *p* < .001, rank-biserial correlation = −0.684). In contrast, when the lead and trail legs crossed the obstacle, there were no significant differences in the normalized MoS between children and adults (*p* > 0.05). A one-sample t test revealed that some children had smaller normalized MoS values when the lead leg (one 2-year-old, one 3-year-old, and two 4-year-olds) and the trail leg crossed the obstacle (one 2-year-old, one 3-year-old, one 4-year-old, and one 5-year-old) than adults (*p* < 0.05).

### 3.4. BoS-CoM distance and CoM velocity

**Figure 6:**
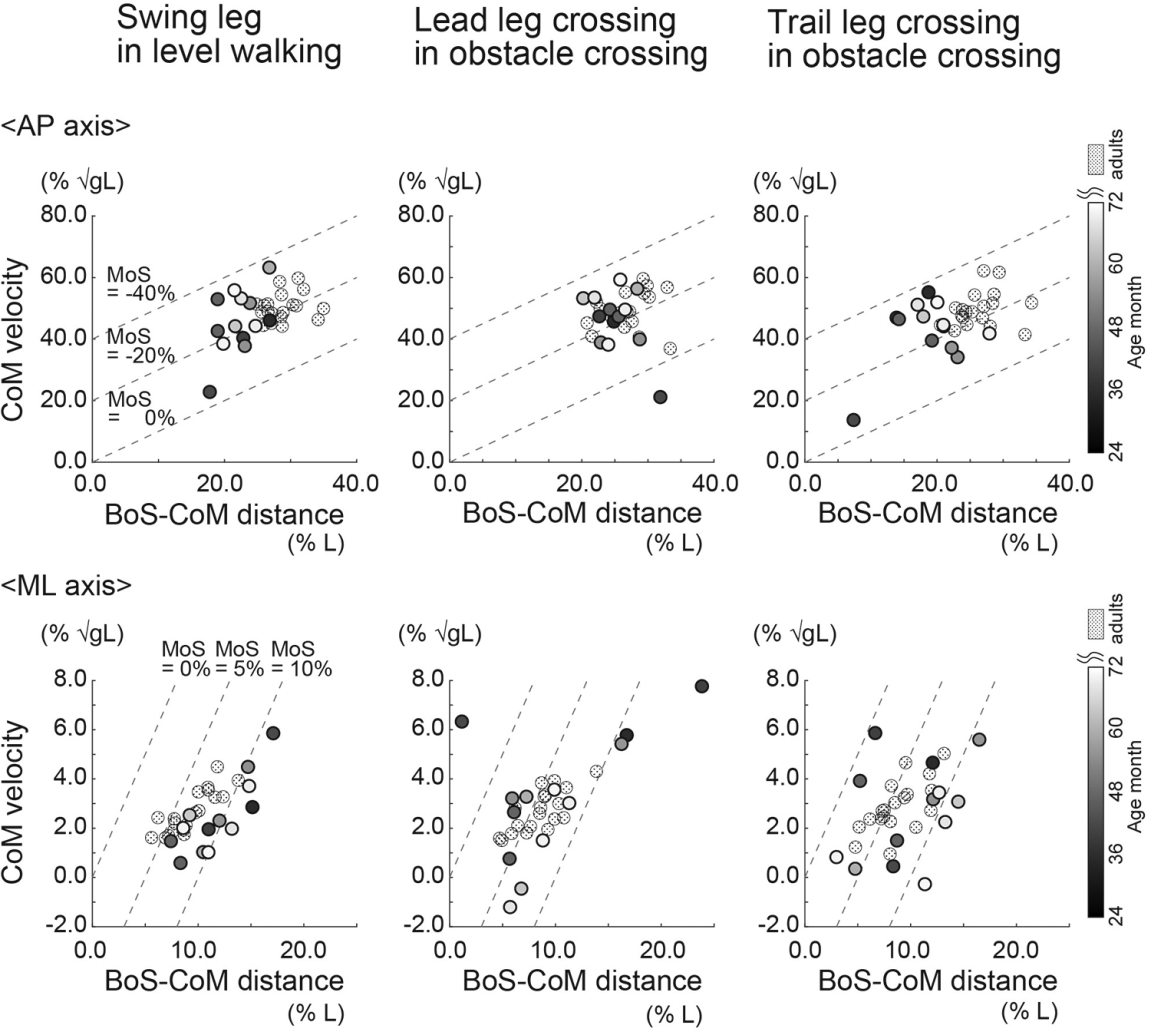
Normalized base of support (BoS)-center of mass (CoM) distance and CoM velocity in the anteroposterior (AP) and mediolateral (ML) directions during level walking and obstacle crossing (with the lead and trail leg, separately). The BoS-CoM distance was normalized to leg length (*L*). The CoM velocity was normalized to the root square of gravity (*g*) × *L*. Circles indicate the mean value of each participant.

Figure 6 shows the normalized BoS-CoM distance and normalized CoM velocity in the AP and ML directions. Along the AP axis, adults showed significantly greater normalized BoS-CoM distances during level walking and after the trail leg crossed the obstacle than children (level walking: *W* = 235.000, *p* < .001, rank-biserial correlation = 0.903; obstacle crossing with trail leg: *W* = 228.000, *p* < .001, rank-biserial correlation = 0.846). There was no significant difference in the normalized BoS-CoM distance between children and adults after the lead leg crossed the obstacle (*p* > 0.05). In terms of the normalized CoM velocity along the AP axis, there were no significant group differences during level walking and after the lead leg crossed the obstacle. In contrast, children had a smaller normalized CoM velocity after the trail leg crossed the obstacle than adults (*W* = 176.000, *p* = 0.045, rank-biserial correlation = 0.425).

Along the ML axis, the normalized BoS-CoM distance during level walking was significantly greater in children than in adults (*W* = 65.000, *p* = 0.024, rank-biserial correlation = −0.474); there were no significant group differences in the normalized BoS-CoM distance when the lead and trail leg crossed the obstacle (*p* > 0.05). Children with a smaller normalized MoS in the ML axis than adults when the lead and trail leg crossed the obstacle showed a significantly smaller normalized BoS-CoM distance than adults (*p* < 0.05). Regarding the normalized CoM velocity along the ML axis during level walking and obstacle crossing, there were no significant differences between children and adults (*p* > 0.05).

### 3.5. Vertical foot clearance, maximum toe height, swing time

**Figure 7:**
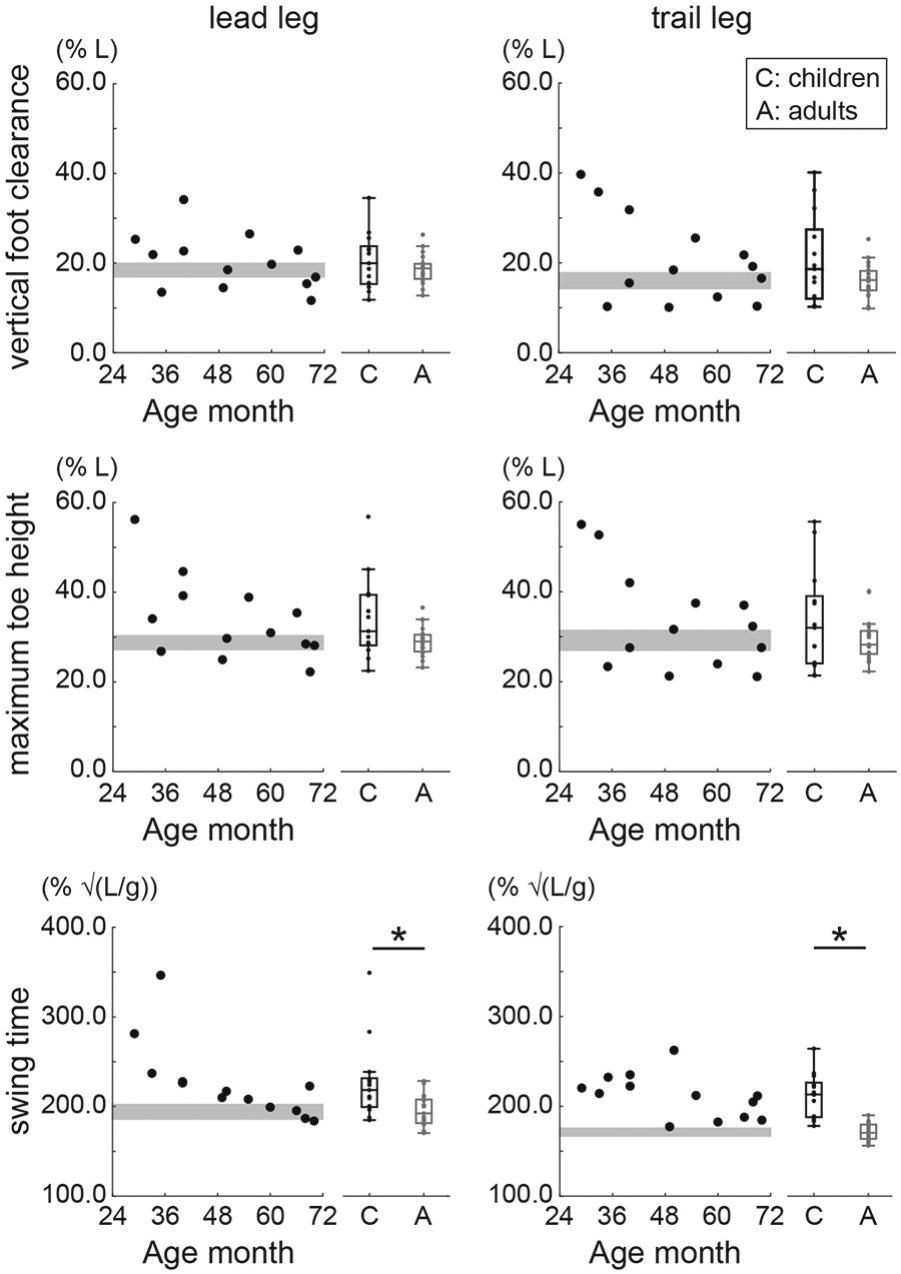
Normalized vertical foot clearance, maximum toe height, and swing time during obstacle crossing. Circles indicate the mean value of within participants. The gray area shows the 95% confidence interval (CI) of the mean of adults. * Indicates *p* < 0.05.

There were no significant differences in the normalized vertical foot clearance and the normalized maximum toe height of the lead and trail legs (*p* > 0.05) (Figure 7). The normalized maximum toe height of the lead and trail legs tended to be greater in younger children. In contrast, the normalized swing time of the lead and trail legs was significantly greater in children than in adults (lead leg: *W* = 54.000, *p* = 0.007, rank-biserial correlation = −0.563; trail leg: *W* = 10.000, *p* < . 001, rank-biserial correlation = −0.919).

### 3.6. Change ratio in gait patterns from obstacle crossing to level walking

**Figure 8:**
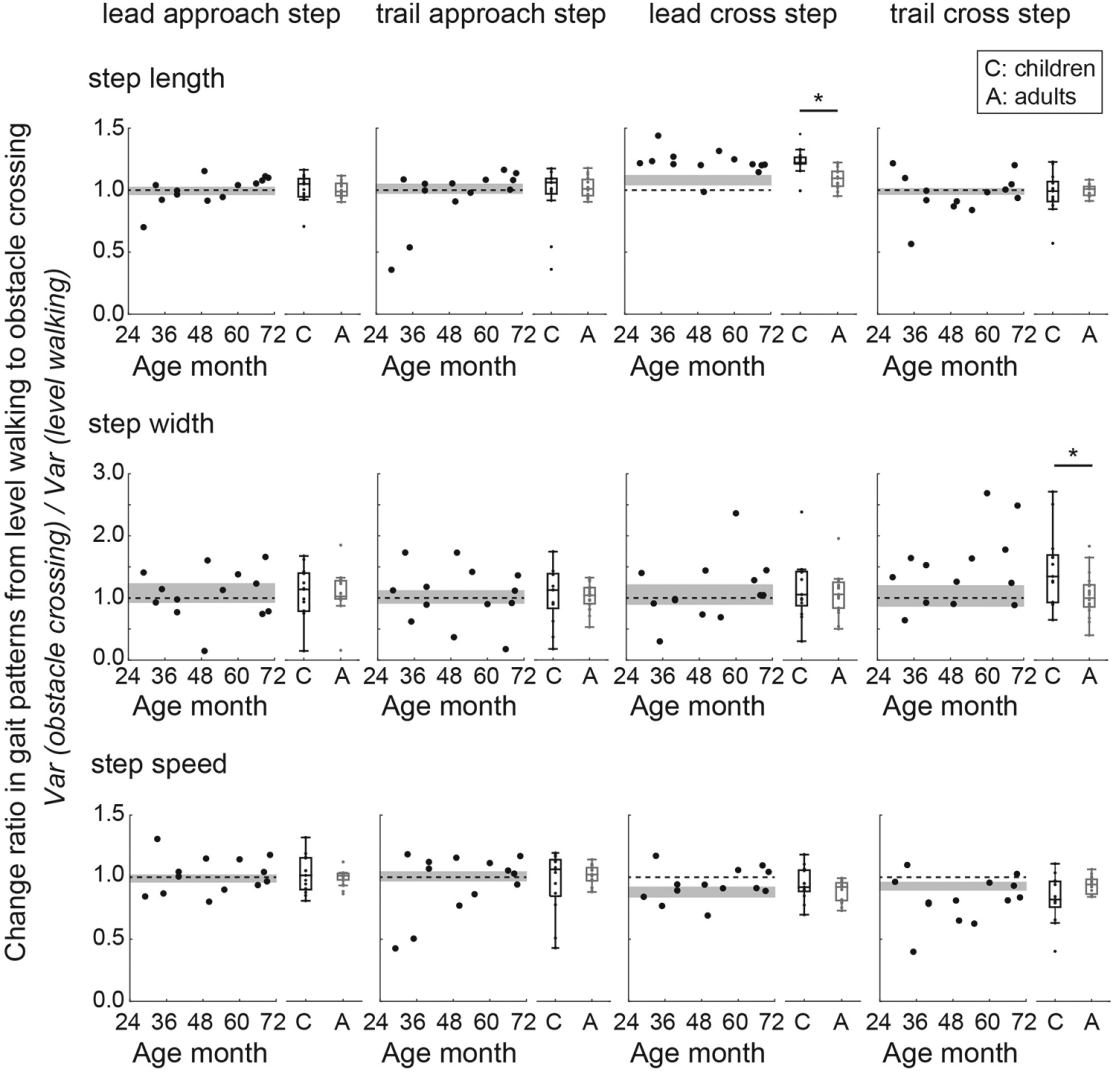
Change ratios of the step length, step width, and step speed from level walking to obstacle crossing. A ratio value greater than 1 indicates that the gait parameter during obstacle crossing was greater than that during level walking. Circles indicate the mean value of within participants. The gray area shows the 95% confidence interval (CI) of the mean of adults. The dotted line indicates a change ratio equal to 1. * Indicates *p* < 0.05.

Children and adults took a longer lead cross step in the obstacle-crossing task than during the level-walking task, as the change ratio of the step length of the lead cross step was significantly greater than 1 (*p* < 0.05) (Figure 8). Additionally, the change ratio of the step length of the lead cross step was significantly larger in children than in adults (*W* = 24.000, *p* < .001, rank-biserial correlation = −0.806). Specifically, two children aged 2 years took a significantly shorter step during the trail approach step than adults (*p* < 0.05). The change ratio of the step width of the trail cross step was significantly greater in children than in adults (*W* = 67.000, *p* = 0.030, rank-biserial correlation = −0.457). There were no significant differences in the change ratios of the step speed of the lead approach step, trail approach step, lead cross step, or trail cross step between children and adults (*p* > 0.05). On the other hand, in the two children aged 2 years, the change ratio of the step speed during the trail approach step was approximately 0.5, which was significantly smaller than that in adults (*p* < 0.05).

## 4. Discussion

The purpose of this study was to investigate dynamic stability during level walking and obstacle crossing in preschool children. In the forward direction, children showed large intraindividual variability in the MoS at the instant of the heel contact; however, they consistently strived to ensure the MoS by increasing the step length of the lead leg during obstacle crossing to a greater extent than adults. In the lateral direction, children exhibited more dynamic stability during level walking than adults, although some children exhibited less dynamic stability, as shown by a small BoS-CoM distance.

First, we evaluated developmental changes in gait patterns during level walking. Previous level-walking studies have reported that the step length and step speed (normalized to body size) become adult-like at approximately 4–5 years of age; in contrast, the normalized step width of preschool children has not yet fully matured (4–8). Our study showed that the step length and step speed (normalized to leg length) developed from 2 to 4 years of age, at which point at 4 years of age, these parameters were the same as those of adults. Although in the present study the normalized step length and the normalized step speed in 5-year-olds were 5% and 2% less than those of adults, respectively, these results were validated because in children between 4 and 13 years of age, these parameters are within ± 4%–5% of those of adults (8). In contrast, the normalized step widths of 2-year-olds and 3–5-year-olds were 12% and 4% larger, respectively, than those in adults, which is supported by a previous study (6). In addition, since the gait parameters calculated in our study fall within the range of values reported in previous studies (4, 8, 30), the children in this study were confirmed to exhibit typical development.

Second, we evaluated the characteristics of obstacle-crossing behavior in preschool children. Preschool children successfully crossed the obstacle of height of 10% of leg length without support. Younger children crossed the obstacle more carefully to avoid falling, as evidenced by obviously decreased gait speed just before crossing obstacles in 2-year-olds and tendency of the increasing maximum toe height with younger age. As reported by Cappellini et al. (23), stopping or reducing gait speed just before an obstacle, as observed in 2-year-olds, clearly differed from the behavior of children aged 3–5 years. Their results suggest that 2-year-olds experienced difficulty in adjusting their foot placement prior to obstacles compared to older children and adults (31). Cautious behavior defined as slow gait speed and great maximum toe height observed in only 2-year-olds might suggest that the complex information processing necessary for obstacle crossing rapidly develops after the age of 2 years.

Children aged 2–5 years attempted to increase forward MoS by increasing the BoS-CoM distance after the lead leg crossed the obstacle. Specifically, one 2-year-old showed a positive forward MoS at the instant of the heel contact of the lead leg. Similarly, a previous obstacle-crossing study that targeted community-dwelling elderly individuals (aged 55–83 years) showed that the MoS at the instant of the heel contact of the lead leg increased in the positive direction (32). The forward CoM momentum should be reduced after the lead leg crosses an obstacle because the trail foot needs to be pulled up vertically (rather than horizontally) in the first half of the swing phase of the trail leg. The increase in the step length of the lead leg resulted in an increase in BoS-CoM distance. A large step length clearly contributed to a great forward MoS in the positive direction (33). In our study, children increased the step length of the lead leg during the obstacle-crossing task compared to the level-walking task to a greater extent than adults. We interpreted this finding as the children attempting to avoid colliding with the obstacle. Moreover, increasing the forward dynamic stability before the trail leg crosses an obstacle may prevent the trail leg from hitting the obstacle. Contact occurs more frequently with the trail leg because of the lack of visual information (34). Therefore, for preschool children, who have a high incidence of tripping and falls, improving the forward dynamic stability at the instant of the heel contact of the lead leg is beneficial.

Children exhibited greater lateral MoS than adults during level walking, whereas some children exhibited lower lateral stability than adults during obstacle crossing. Among the thirteen preschoolers, seven children had more than 1 trial with a negative lateral MoS. In level walking, it has been reported that the normalized ML MoS during level walking in preschool children was greater than that in older children aged 6–10 years (6). Additionally, our results suggest that normalized ML MoS in preschool children was greater than that in adults. In the obstacle crossing, however, it suggests that the ML MoS in preschool children tended to be small compared to adults. The loss of lateral MoS was attributed to the reduced BoS-CoM distance from the level-walking task to the obstacle-crossing task. The task-related change in the CoM velocity was small in this study. The decreased BoS-CoM distance in preschoolers might accommodate a longer swing duration than in adults. A long swing duration shifts the CoM closer to the stance leg to stabilize the CoM (35), which exposes the CoM to the risk of falling toward the supporting leg side. Limited hip adductor muscle, one of the contributions of frontal plane stability during a single stance phase (36), might also promote dynamic instability during obstacle crossing. Large individual differences in the ML MoS during obstacle crossing were observed in preschool children. Since the present study involved preschoolers of different ages at different developmental stages of gait stability, it is not surprising that there was greater between-participant variability. In addition, given the small number of trials per subject, the observed individual differences might be attributed to large trial-to-trial variability. Although the present study was not designed to quantify the variability in MoS, it has been reported that a large within-participant variability in MoS is also related to a greater risk of fall and instability (37). Future studies may measure the variability in MoS to reveal the nature of stability during obstacle crossing in children.

Only preschool children had a wider trail-leg step than adults during obstacle crossing compared to level walking, which might be interpreted as a strategy of recovering lateral dynamic stability. A large step width contributed to a large lateral MoS (38, 39). Previous obstacle-crossing studies have reported that children aged over 7 years take wide steps when the lead and trail legs cross obstacles and that the step width was greater at the instant of the heel contact of the trail leg than the lead leg (29, 40). Therefore, it appears that even preschool children can recover lateral dynamic stability by adjusting foot placement along the ML axis.

Excessive foot elevation prevents contact with obstacles but might cause loss of lateral dynamic stability. According to a previous study, excessive foot elevation results in low control of the ML CoM position (13). In the present study, the normalized maximum toe height tended to be greater with decreasing age, and that in younger children was greater than that in adults. In addition, Snapp-Childs and Bingham (15) reported that children aged 3–6 years exhibited a large foot clearance to cope with the self-constraints of high foot-movement variability at that age. Moreover, to ensure the same absolute foot clearance as adults when crossing height-normalized obstacles, children need to lift their feet higher than adults because their body size is smaller. Attempting to produce the same foot clearance as adults might cause a loss of lateral dynamic stability.

Reduced walking speed increases dynamic stability in the forward direction but not in the lateral direction. In the forward direction, reduced walking speed was previously found to provide greater MoS than increased step length, as changes in walking speed had a greater impact on the MoS than changes in the step length when the walking speed and step length were varied independently (33). Conversely, in the ML direction, decreased walking speed was attributed to a large ML CoM displacement and a large ML CoM velocity, which resulted in a decreased lateral MoS (41). Similar to Cappellini et al. (23), in the present study, 2-year-olds significantly reduced their step speed and step length just before crossing the obstacle. Thus, 2-year-olds secured dynamic stability in the forward direction by significantly reducing the step speed just before crossing the obstacle at the expense of dynamic stability in the lateral direction.

The present study showed that 2–5-year-old children have already acquired the ability of self-obstacle-crossing, but only under the condition of obstacles at a height of 10% of leg length (i.e., 4–6 cm). If preschool children are faced with higher obstacles, they may attempt to step over obstacles that are not appropriate for their body size. It has been reported that preschool children (3–5 years) make large errors when making decisions about whether to reach through openings of different sizes relative to their body size (42). Models with rigid link segments and inverted pendulums have some limitations. We calculated the CoM position using anthropometric data from children reported by Jensen et al. (27), which were obtained from boys aged 4–15 years. In the present study, the 13 preschoolers included 5 toddlers aged 2–3 years and 5 girls. In addition, the estimation of pendulum length, calculated by leg length × 1.24, applies well to adults but may not apply to children. In other words, since toddlers have a greater head mass proportion than adults, l was estimated to be smaller. If l is larger, the MoS will be smaller. However, the suggestion that preschoolers were likely to be dynamically instable in the lateral direction during obstacle crossing was not overturned.

## 5. Conclusion

Children aged 2–5 years attempted to maintain the MoS in the forward direction by increasing the step length at the instant of the heel contact of the lead leg in the lateral direction, while some preschoolers exhibited dynamic instability when crossing obstacles related to a small BoS-CoM distance. Younger children crossed the obstacle more carefully to avoid falls in the forward direction, as evidenced by obviously decreased gait speed just before crossing obstacles in 2-year-olds and increases in foot elevation with younger age.

## Data Availability

The raw data supporting the conclusions of this article will be made available by the authors, without undue reservation.
