# Supplementary figures and images for: Dynamic stability during level walking and obstacle crossing in children aged 2–5 years estimated by marker-less motion capture
Source: Front Sports Act Living. 2023 Apr 6;5:1109581. doi: 10.3389/fspor.2023.1109581 (PMC10116057; doi:10.3389/fspor.2023.1109581)

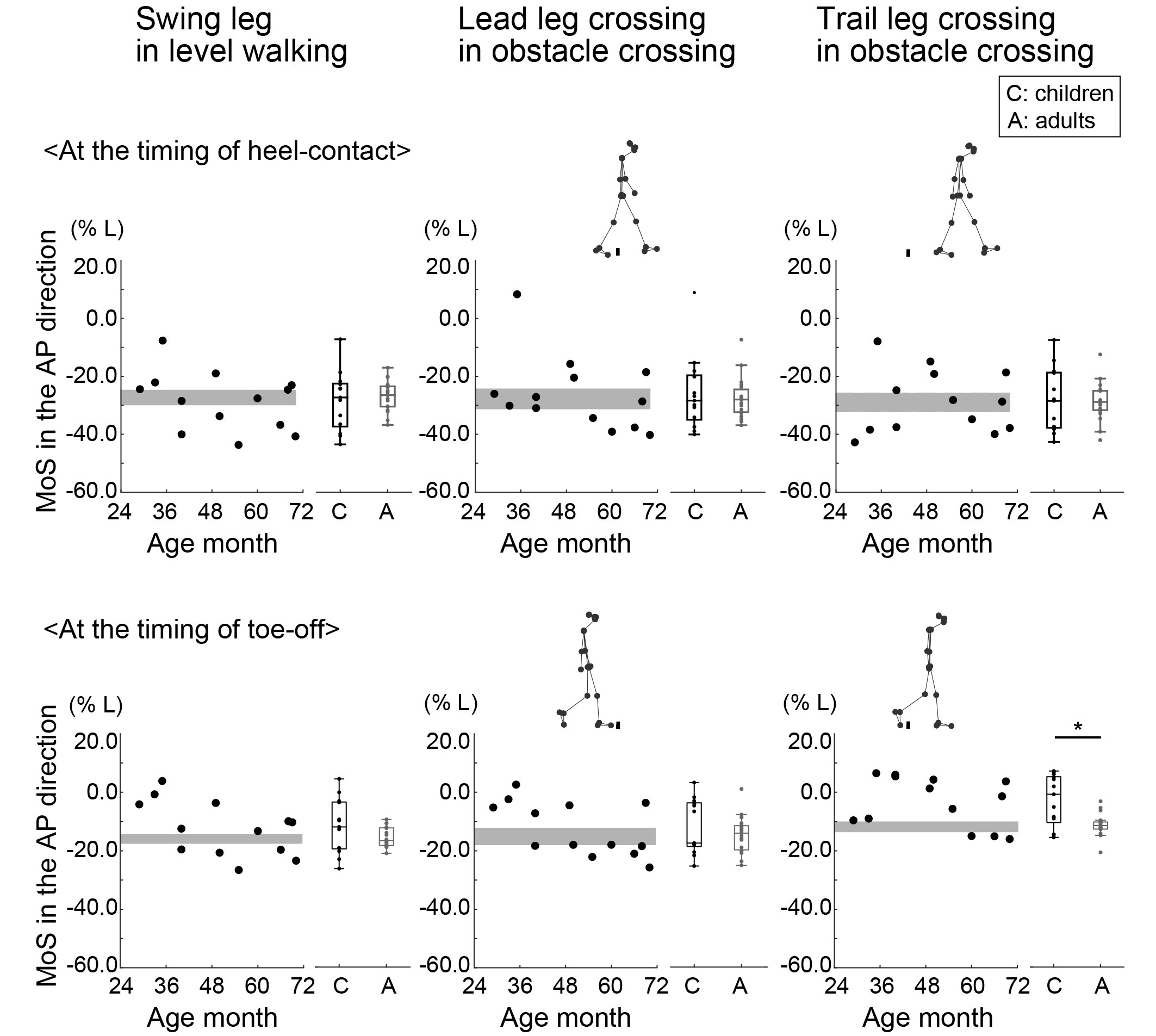

Supplement: Supplementary file 1 [file Image1.jpeg]
